# An Integrated Approach for Making Inference on the Number of Clusters in a Mixture Model

**DOI:** 10.3390/e21111063

**Published:** 2019-10-30

**Authors:** Erlandson Ferreira Saraiva, Adriano Kamimura Suzuki, Luis Aparecido Milan, Carlos Alberto de Bragança Pereira

**Affiliations:** 1 Instituto de Matemática, Universidade Federal de Mato Grosso do Sul, Campo Grande 79070-900, Brazil; cpereira@ime.usp.br; 2 Departamento de Matemática Aplicada e Estatística, Universidade de São Paulo, São Carlos 13566-590, Brazil; suzuki@icmc.usp.br; 3 Departamento de Estatística, Universidade Federal de São Carlos, São Carlos 13565-905, Brazil; dlam@ufscar.br; 4 Instituto de Matemática e Estatística, Universidade de São Paulo, São Paulo 05508-090, Brazil

**Keywords:** model-based clustering, mixture model, EM algorithm, integrated approach

## Abstract

This paper presents an integrated approach for the estimation of the parameters of a mixture model in the context of data clustering. The method is designed to estimate the unknown number of clusters from observed data. For this, we marginalize out the weights for getting allocation probabilities that depend on the number of clusters but not on the number of components of the mixture model. As an alternative to the stochastic expectation maximization (SEM) algorithm, we propose the integrated stochastic expectation maximization (ISEM) algorithm, which in contrast to SEM, does not need the specification, a priori, of the number of components of the mixture. Using this algorithm, one estimates the parameters associated with the clusters, with at least two observations, via local maximization of the likelihood function. In addition, at each iteration of the algorithm, there exists a positive probability of a new cluster being created by a single observation. Using simulated datasets, we compare the performance of the ISEM algorithm against both SEM and reversible jump (RJ) algorithms. The obtained results show that ISEM outperforms SEM and RJ algorithms. We also provide the performance of the three algorithms in two real datasets.

## 1. Introduction

Recently, there has been increasing interest in modeling using mixture models. This is mainly due to the flexibility for treating heterogeneous populations. Under a data-clustering framework, this model has the advantage of being probabilistic, and then the obtained clusters can have a better interpretation from a statistical point of view [1]. This contrasts with usual methods, such as k-means or hierarchical clustering, in which clusters are not statistically based, as discussed by [2].

From a frequentist viewpoint, the standard method to get the maximum likelihood estimates for the parameters of a mixture model is based on the use of the Expectation Maximization (EM) algorithm [3]. However, for the use of this algorithm, the number of components *k* of the mixture model needs to be known a priori. As the resulting model is highly dependent on the choice of this value, the main question is how to set the *k* value. Selecting an erroneous *k* value may cause the non-convergence of the algorithm and/or a low exploration of the clusterings. In addition, depending on the *k* value chosen we may have empty components, and therefore, there are no maximum likelihood estimates for these components.

An approach frequently used to determine the best *k* value among a fixed set of values is the use of the stochastic version of the EM algorithm (SEM) with some model selection criterion, such as the Akaike information criterion (AIC) [4,5] or the BIC [6]. In this approach, models are fitted for a set of predefined *k* values, and the best model is the one that has the smallest AIC or BIC value.

However, as discussed by [7], to adjust several models for a predefined set of values for the number of the cluster and compare them using some model selection criterion is not a practical and efficient procedure. Therefore, it is desirable to have an efficient algorithm to calculate the optimal number of clusters together with the estimation of the parameters of each mixture component. In this scenario, the Bayesian approach was successfully performed considering the Markov chain Monte Carlo (MCMC) algorithm with reversible jumps, described by [8] in the context of univariate normal mixture models. On the other hand, a difficulty often encountered in implementing a reversible jump algorithm (RJ) is the construction of efficient transition proposals that lead to a reasonable acceptance rate.

Following in the line of MCMC algorithms, [9] proposes a split–merge MCMC procedure for the conjugated Dirichlet process mixture model using a restricted Gibbs sampling scan to determine a split proposal, where the number of scans (tuning parameter) must be previously fixed by the user, and [10] extend their method to a nonconjugated Dirichlet process mixture model. [11] proposes a data-driven split-and-merge approach. In this proposal, the number of clusters is updated according to the creation of a new component based on a single observation and using a split–merge strategy, developed based on the use of the Kullback–Leibler divergence. A difficulty encountered for implementing this algorithm is the obtaining of the mathematical expression for the Kullback–Leibler divergence, which does not always have known analytical expression. In addition, the sequential allocation used in the split–merge strategy of these three works may make the algorithm slow when the sample size is great, and the computation implementation of these methods is not so simple.

The present work proposes an integrated approach that, in a joint way, selects the number of clusters and estimates the parameters of interest. With this approach, the mixture weights are integrated out to obtain allocation probabilities that depend on the number of clusters (nonempty components) but do not depend on the number of components *k*. In addition, considering *k* tending to infinity, this procedure introduces a positive probability of a new cluster being created by a single observation. When a new cluster is created, the parameters associated with it are generated from its posterior distribution. We then developed the ISEM (integrated stochastic expectation maximization) algorithm to estimate the parameters of interest. This algorithm configures a setting for latent allocation variables c according to allocation probabilities, and then the cluster parameters are updated conditionally on c as follows: for clusters with at least two observations, the parameter values are the maximum likelihood estimates; for the clusters with only one observation, the parameter values are generated from their posterior distribution.

In order to illustrate the computation implementation of the method and verify its performance, we have considered a specific model in which data are generated from mixtures of univariate normal distributions. This model allows us to avoid the label switching problem by considering the labeling of the components according to the increasing order of the component averages, as done by [8,11,12,13], among others. But we emphasize that our algorithm is not restricted to this particular model. For instance, for the multivariate case, we may consider the labeling of the components according to the eigenvalues of the current covariance matrix, as done by [14]. However, a detailed discussion of the multivariate case will be done in a future paper.

We also compare the performance of the ISEM with both SEM and RJ algorithms. The criteria used to compare the methods are the estimated probability of the number of clusters, convergence of the sampled values, mixing, autocorrelation, and computation time. We also applied the three algorithms to two real datasets. The first is the well-known Galaxy data, and the second is a dataset on Acidity.

The remainder of the paper is as follows. Section 2 describes the mixture model and the estimation process based on the SEM algorithm. Section 3 develops the integrated approach and describes the ISEM algorithm. Section 4 shows how we applied the algorithm to simulated datasets in order to assess its performance. Section 5 describes the application of the three algorithms to two real datasets. Section 6 is about our final remarks. Additional details are in the Supplementary Material, which is referred to as “SM” in this paper. Table 1 presents the main notations used throughout the article.

## 2. Mixture Model and SEM Algorithm

Let $y=(y_{1},\ldots ,y_{n})$ be a vector of independent observations from a mixture model with *k* components, i.e.,

(1) $$f\left(y_{i}|w,\theta_{k},k\right)=\sum_{j=1}^{k}w_{j}f\left(y_{i}|\theta_{j}\right),$$

where $f(y_{i}|\theta_{j})$ is the density of a family of parametric distributions with parameters $\theta_{j}$ (scalar or vector), $\theta_{k}=\left(\theta_{1},\ldots ,\theta_{k}\right)$ are the parameters of the components, and $w=(w_{1},\ldots ,w_{k})$, $w_{j}>0$ and $\sum_{j=1}^{k}w_{j}=1$ are component weights.

The log-likelihood function for $\left(\theta_{k},w\right)$ is given by

$$l\left(\theta_{k},w|y,k\right)=\log\left\{\prod_{i=1}^{n}\left[\sum_{j=1}^{k}w_{j}f\left(y_{i}|\theta_{j}\right)\right]\right\}=\sum_{i=1}^{n}\log\left\{\left[\sum_{j=1}^{k}w_{j}f\left(y_{i}|\theta_{j}\right)\right]\right\}.$$

The mathematical notation $l(\theta_{k},w|y,k)$ is given as in the book of Casella and Berger (2002).

The usual procedure to obtain the maximum likelihood estimators consists of getting partial derivatives of $l(\theta_{k},w|y)$ in relation to $\theta_{j}$ and then equalizing the result to zero, i.e.,

(2) $$\begin{array}{c} \frac{dl(\theta_{k},w|y)}{d\theta_{j}}=\sum_{i=1}^{n}\frac{w_{j}f\left(y_{i}|\theta_{j}\right)}{\sum_{j=1}^{k}w_{j}f\left(y_{i}|\theta_{j}\right)}\frac{d\log\left[f(y_{i}|\theta_{j})\right]}{d\theta_{j}}=0, \end{array}$$

for j=1,…,k.

But, note that in (2), the maximization procedure consists of a weighted maximization process of the log-likelihood function with each observation $y_{i}$ having a weight associated to component *j* given by

(3) $$w_{ij}^{*}=\frac{w_{j}f\left(y_{i}|\theta_{j}\right)}{\sum_{j=1}^{k}w_{j}f\left(y_{i}|\theta_{j}\right)},$$

for i=1,…,n and j=1,…,k. However, these weights depend on the parameters that we are trying to estimate. In this way, we cannot obtain a “closed” mathematical expression that allows the direct maximization of the log-likelihood function. Due to this, the mixture problem is reformulated as a complete-data problem [12,15].

### Complete-Data Formulation

Consider associated to each observation $y_{i}$ a latent indicator variable $c_{i}$ not known, so that if $c_{i}=j$, then $y_{i}$ is from component *j*, for i=1,…,n and j=1,…,k. The probability of $c_{i}=j$ is $w_{j}$, $P\left(c_{i}=j|w,k\right)=w_{j}$, for i=1,…,n and j=1,…,k. Letting $n_{j}$ be the number of observations from component *j* (i.e., the number of $c_{i}$s equals to *j*), the joint probability for $c=(c_{1},\ldots ,c_{n})$ given w and *k* is

(4) $$\pi \left(c|w,k\right)=\underset{j=1}{\prod^{k}}w_{j}^{n_{j}}.$$

The distribution of the number of observations assigned to each component, $n_{1},\ldots ,n_{k}$, called the occupation number, is multinomial, $\left(n_{1},\ldots ,n_{k}|n,w\right)∼Multinomial\left(n,w\right)$, where $n=n_{1}+\ldots +n_{k}$.

Thus, under this augmented framework, we have that

(1) the probability of $c_{i}=j$, conditional on observation $y_{i}$ and on component parameters $\theta_{k}$, is $w_{ij}^{*}$, i.e., $P\left(c_{i}=j|y_{i},\theta_{k},k\right)=w_{ij}^{*}$, for $w_{ij}^{*}$ given in Equation (3), for i=1,…,n and j=1,…,k. That is, although the indicator variables are nonobservable, they are implicitly present in the estimation procedure given in Equation (2).
(2) the log-likelihood function for $\left(\theta_{k},w\right)$, conditional on complete data (y,c), is given by
  $$\begin{array}{c} l\left(\theta_{k},w|y,c\right)=\log\left\{\prod_{j=1}^{k}w_{j}^{n_{j}}L\left(\theta_{j}|y\right)\right\}=\sum_{j=1}^{k}\left[n_{j}\log\left(w_{j}\right)+l\left(\theta_{j}|y\right)\right], \end{array}$$
  where $l\left(\theta_{j}|y\right)=\log\left[L(\theta_{j}|y)\right]$ is the log-likelihood function for component *j*, for j=1,…,k. Thus, the estimation procedure of the *k* component parameters reduce to *k* independent problems of estimation. For example, for a normal mixture model, the maximum likelihood estimates for component parameters $\theta_{j}=\left(\mu_{j},\sigma_{j}^{2}\right)$ is ${\hat{\theta }}_{j}=\left({\hat{\mu }}_{j},{\hat{\sigma }}_{j}^{2}\right)=\left({\bar{y}}_{j},s_{j}^{2}\right)$, where ${\bar{y}}_{j}$ and $s_{j}^{2}$ are, respectively, the average and variance of the observations allocated to component *j*, for j=1,…,k.

From this complete-data formulation, the estimation procedure is given by an iterative process with two steps. In the first one, the allocation indicator variables are updated conditional on component parameters, and in the subsequent step, the component parameters are updated conditional on configuration of the allocation indicator variables.

The usual algorithm used to implement these two steps is the EM algorithm [3]. The stochastic version of the EM algorithm (SEM) can be implemented according to Algorithm 1.

**Algorithm 1** SEM Algorithm1: Initialize the algorithm with a configuration $c^{\left(0\right)}=\left(c_{1}^{\left(0\right)},\ldots ,c_{n}^{\left(0\right)}\right)$ for allocation indicator variables.2: **procedure** For the *s*-th iteration of the algorithm, s=1,…
3:     get the maximum likelihood estimates ${\hat{\theta }}_{k}^{\left(s\right)}=\left({\hat{\theta }}_{1}^{\left(s\right)},\ldots ,{\hat{\theta }}_{k}^{\left(s\right)}\right)$ and ${\hat{w}}^{\left(s\right)}=\left({\hat{w}}_{1}^{\left(s\right)},\ldots ,{\hat{w}}_{k}^{\left(s\right)}\right)$         conditional on configuration $c^{\left(s-1\right)}$;4:     **if**
$\left|\frac{l\left({\hat{\theta }}_{k}^{\left(s\right)},{\hat{w}}^{\left(s\right)}|y\right)-l\left({\hat{\theta }}_{k}^{\left(s-1\right)},{\hat{w}}^{\left(s-1\right)}|y\right)}{l\left({\hat{\theta }}_{k}^{\left(s-1\right)},{\hat{w}}^{\left(s-1\right)}|y\right)}\right|<\epsilon$, where ϵ is a threshold value previously fixed, **then** stop the algorithm. Otherwise, go to item (iii);5:     conditional on ${\hat{\theta }}_{k}^{\left(s\right)}$ and ${\hat{w}}^{\left(s\right)}$, update $c=(c_{1},\ldots ,c_{n})$ as follows. For i=1,…,n and j=1,…,k do the following:6:     Let $z_{i}=\left(z_{i1},\ldots ,z_{ik}\right)$ be a indicator vector, so that $z_{ij}=0$ or $z_{ij}=1$;7:     Generate $z_{i}∼Multinomial\left(1,w_{i}^{*}\right)$, where $w_{i}^{*}=\left(w_{i1}^{*},\ldots ,w_{ik}^{*}\right)$ and $w_{ij}^{*}$ is obtained from Equation (3) doing $\theta_{j}={\hat{\theta }}_{j}$ and $w_{j}={\hat{w}}_{j}$. If $z_{ij}=1$, then do $c_{i}=j$;8:     Do s=s+1 and return to step (3).

Although it is simple to implement computationally, the SEM algorithm may present some practical problems. As discussed by [16], the algorithm may present a slow convergence. Due to this, some authors, such as [17,18], discuss how to set up the start values in order to increase the convergence. In addition, [15] discusses the non-existence of the global maximum estimator.

Moreover, in this algorithm, the *k* value must be known previously. For the cases in which *k* is an unknown quantity, the best *k* value is chosen by fitting a set of models associated with a set of predefined *k* values and comparing them according to AIC [4,5] or BIC [6] criteria. Furthermore, given a sample of size *n* and fixed a *k* value, there exists a positive probability, given by ${\left(1-w_{j}\right)}^{n}\neq 0$, of the *j*-th component not having observations allocated in an iteration of the algorithm. In this case, we have an empty component, and the maximum likelihood estimates cannot be calculated for these components. Thus, in order to avoid the practical problems presented by the EM algorithm, we propose an integrated approach.

## 3. Integrated Approach

We start our integrated approach linking data clustering to a mixture model. For this, consider a sampling process from a heterogeneous population that is subdivided into *k* sub-populations. Thus, it is natural to assume that the sampling process consists of the realization of the following steps:

(i) choose a sub-population *j* with probability $w_{j}$, where $w_{j}$ is the relative frequency of the *j*-th sub-population in relation to the overall population;
(ii) sample a $Y_{i}$ value of this sub-population,

for i=1,…,n and j=1,…,k, where *n* is the sample size.

Let $\left(Y_{i},c_{i}\right)$ be a sample unit, where $c_{i}$ is an indicator allocation variable that assumes a value of the set {1,…,k} with probabilities $\left\{w_{1},\ldots ,w_{k}\right\}$, respectively. Thus, assuming that subpopulation *j* is modeled by a probability distribution $F(\theta_{j})$ indexed by parameter $\theta_{j}$ (scalar or vector), we have that

$$\left(Y_{i}|c_{i}=j,\theta_{j}\right)∼F\left(\theta_{j}\right)\;\;\;\mathrm{and}\;\;\;P\left(c_{i}=j|w\right)=w_{j},$$

for i=1,…,n and j=1,…,k.

However, in clustering problems, the $c_{i}$’s values are non-observable. Thus, the probability of $c_{i}=j$ is $w_{j}$, and the marginal probability density function for $Y_{i}=y_{i}$ is given by Equation (1).

In addition, as the model in Equation (1) is a population model; so there exists a non-null probability ${\left(1-w_{j}\right)}^{n}$ that the *j*-th component is an empty component. Thus, the number of clusters (i.e., non-empty components) is smaller than the number of components *k*. As viewed in the description of the EM algorithm, the number of clusters is defined by the configuration of the latent allocation variables c; thus hereafter, we will denote the number of clusters by $k_{c}$, for $k_{c}\leq k$.

Since the interest lies in the configuration of c, let us marginalize out the weights of the mixture model. Thus, integrating density (4) with respect to the prior $Dirichlet\left(\frac{\gamma }{k},\ldots ,\frac{\gamma }{k}\right)$ distribution of the weights, denoted by $\left(w_{1},\ldots ,w_{k}\right)|k,\gamma ∼Dirichlet\left(\frac{\gamma }{k},\ldots ,\frac{\gamma }{k}\right)$, the joint probability for c is given by (see Appendix 3 of the SM)

(5) $$\pi \left(c|\gamma ,k\right)=\frac{\Gamma (\gamma )}{\Gamma (n+\gamma )}\prod_{j=1}^{k}\frac{\Gamma (n_{j}+\frac{\gamma }{k})}{\Gamma \left(\frac{\gamma }{k}\right)}.$$

Similarly, the conditional probability for $c_{i}=j$ given $c_{-i}=\left(c_{1},\ldots ,c_{i-1},c_{i+1},\ldots ,c_{n}\right)$, is given by

(6) $$\pi \left(c_{i}=j|c_{-i},\gamma ,k\right)=\frac{n_{j,-i}+\frac{\gamma }{k}}{n+\gamma -1},$$

where $n_{j,-i}$ is the number of observations allocated to the *j*-th component, excluding the *i*-th observation, for i=1,…,n and j=1,…,k.

As the main interest is in $k_{c}$ and not *k*, we remove *k* from Equation (6) by letting *k* tend to infinity. Under this assumption, the probability reaches the following limit:

(7) $$\pi \left(c_{i}=j|c_{-i},\gamma \right)=\frac{n_{j,-i}}{n+\gamma -1},$$

when $n_{j,-i}>0$, for i=1,…,n and $j=1,\ldots ,k_{c}$, where $k_{c}$ is the number of clusters defined by configuration c. In addition, we now have a probability of the *i*-th observation being allocated to one of the other infinite components, which is given by

(8) $$\pi \left(c_{i}=j^{*}|c_{-i},\gamma \right)=\frac{\gamma }{n+\gamma -1},$$

for $j^{*}\notin \left\{1,\ldots ,k_{c}\right\}$. This is the probability of the observation $y_{i}$ creating a new cluster, for i=1,…,n. The probabilities in (7) and (8) are equivalent to the update probabilities of a Dirichlet process mixture model. See, for example, [19,20,21].

Given $y_{i}$, the conditional probability for $c_{i}=j$, such that $n_{j,-i}>0$, is

(9) $$\pi_{ij}=\pi \left(c_{i}=j|y_{i},\theta_{j},c_{-i},\gamma \right)=\frac{n_{j,-i}}{n+\gamma -1}f\left(y_{i}|\theta_{j}\right),$$

for i=1,…,n and $j=1,\ldots ,k_{c_{-i}}$, where $k_{c_{-i}}$ is the number of clusters excluding the *i*-th observation. At this point, it is important to note that if an observation $y_{i}$ is allocated to a component *j*, $c_{i}=j$, and $n_{j}>1$, then $n_{j,-i}\geq 1$ and $k_{c_{-i}}=k_{c}$. But if $c_{i}=j$ and $n_{j}=1$, then $n_{j,-i}=0$ and $k_{c_{-i}}=k_{c}-1$.

In order to define the conditional probability of the *i*-th observation creating a new cluster $j^{*}$, we integrate parameters out for this case, for $j^{*}=k_{c_{-i}}+1$. This was done because that probability does not depend on the parameter value $\theta_{j^{*}}$. Thus, the conditional posterior probability for $C_{i}=j^{*}$ is

(10) $$\pi_{ij^{*}}=\pi \left(c_{i}=j^{*}|y_{i},c_{-i},\gamma \right)=\frac{\gamma }{n+\gamma -1}I\left(y_{i}\right),$$

where $I\left(y_{i}\right)=\int f\left(y_{i}|\theta_{j^{*}}\right)\pi \left(\theta_{j^{*}}\right)d\theta_{j^{*}}$ and $\pi (\theta_{j^{*}})$ is the density of the prior distribution for $\theta_{j^{*}}$, for i=1,…,n.

As is known from the literature, the likelihood function for a mixture model is non-identifiable, i.e., any permutation of the components’ labels lead to the same likelihood function (see, for example, [8,11,22,23,24]). Thus, in order to get identifiability, we assume that $\mu_{1},\ldots ,\mu_{k_{c}}$ are the component means for clusters and that $\mu_{1}<\ldots <\mu_{k_{c}}$. However, it does not prevent the algorithm described in the next Section from being applicable to another labeling criterion. Additional discussion about label switching can be found in [22,23].

### Integrated SEM Algorithm

Using probabilities given in Equations (9) and (10), we update the allocation indicator variables according to Algorithm 2.

Conditional on a configuration c, we have $k_{c}$ clusters. So, we update parameters of interest according to Algorithm 3. We then join Algorithms 2 and 3 to get the Algorithm 4.

After the *S* iterations, we discard the first *B* iterations as a burn-in. In the following, we also consider “jumps” of size *h*, i.e., only one draw every *h* is extracted from the original sequence in order to obtain a sub-sequence of size H=(S−B)/h to make inferences. Denote this sub-sequence by S(H).

Consider $N_{k_{c}}\left(j\right)$ to be the number of times that $k_{c}=j$ in S(H), for $j\in \{1,\ldots ,K_{max}\}$, where $K_{max}$ is the maximum $k_{c}$ value sampled in the course of iterations. Thus, $\tilde{P}\left(k_{c}=j\right)=\frac{N_{k_{c}}\left(j\right)}{H}$ is the estimated probability for $k_{c}=j$. We then consider

$${\tilde{k}}_{c}=\underset{1\leq j\leq K_{max}}{argmax}\left(\tilde{P}\left(k_{c}=j\right)\right)$$

as being the estimates for the number of components $k_{c}$.

Appendix 1 of the SM presents the mathematical expression used to determine a configuration for c and estimates for the parameters of the clusters, conditional on the estimate ${\tilde{k}}_{c}$.

**Algorithm 2** Updating c1: Let $c=(c_{1},\ldots ,c_{n})$ be the current configuration for latent allocation variables. Then, update c as follows.2: **procedure** For i=1,…,n:3:     Remove $c_{i}$ from the current state c, obtaining $c_{-i}$ and $k_{c_{-i}}$;4:     Generate a variable $Z_{i}=\left(Z_{i1},\ldots ,Z_{ik_{c}}\right)∼Multinomial\left(1,P_{i}\right),$ where $P_{i}=\left(\pi_{i1},\ldots ,\pi_{ik_{c_{-i}}},\pi_{ij^{*}}\right)$ for $\pi_{ij}$ given in (9) and $\pi_{ij^{*}}$ given in (10), for $j=1,\ldots ,k_{c_{-i}}$ and $j^{*}=k_{c_{-i}}+1$;5:     If $Z_{ij}=1$, for $j\in \{1,\ldots ,k_{c_{-i}}\}$, set up $c_{i}=j$ and do $n_{j}=n_{j,-i}+1$;6:     If $Z_{ij^{*}}=1$, do $n_{j^{*}}=1$ and $k_{c}=k_{c_{-i}}+1$. As this new cluster has just one observation allocated, set as component parameter $\theta_{j^{*}}=\theta_{j}^{g}$, where $\theta_{j}^{g}$ is a value generated from posterior distribution $\pi \left(\theta_{j^{*}}|y_{i}\right)\propto f\left(y_{i}|\theta_{j^{*}}\right)\pi \left(\theta_{j^{*}}\right)$, where $f(y_{i}|\theta_{j^{*}})$ is the probability density function for $y_{i}$ conditional on $\theta_{j^{*}}$ and $\pi (\theta_{j^{*}})$ is the density of the prior distribution for $\theta_{j^{*}}$. Relabel the $k_{c}$ clusters in order to maintain the adjacency condition. If the component mean $\mu_{j^{*}}$ of the new cluster is such that:7:     $\mu_{j^{*}}=\underset{1\leq j\leq k_{c}}{min}\mu_{j}$, then do $j^{*}=1$ and relabel all other clusters doing j+1;8:     $\mu_{j^{*}}=\underset{1\leq j\leq k_{c}}{max}\mu_{j}$, then do $j^{*}=k_{c}$ and keep all other clusters labels; 9:     $\mu_{j}<\mu_{j^{*}}<\mu_{j+1}$, for $j\neq \{1,k_{c}\}$, then do $j^{*}=j+1$ and relabel all other clusters $j^{\prime }\geq j+1$ doing $j^{\prime }=j^{\prime }+1$.

**Algorithm 3** Updating cluster parameters1: Let $\theta_{k_{c}}=\left(\theta_{1},\ldots ,\theta_{k_{c}}\right)$ be the current parameter values of the clusters. Conditional on configuration c, get $\theta_{k_{c}}^{updated}=\left(\theta_{1}^{updated},\ldots ,\theta_{k_{c}}^{updated}\right)$ as follows:2: **if** cluster *j* is such that $n_{j}>1$, **then** do $\theta_{j}^{updated}={\hat{\theta }}_{j}$, where ${\hat{\theta }}_{j}$ are the maximum likelihood estimates of the *j*-th cluster;3: **if** cluster *j* is such that $n_{j}=1$, **then** generate $\theta_{j}^{g}$ from conditional posterior distribution $\pi (\theta |y_{i})$ and set $\theta_{j}^{updated}=\theta_{j}^{g}$;4: Do $\theta_{k}=\theta_{k}^{updated}$ only if the adjacency condition $\mu_{1}^{updated}<\ldots <\mu_{k_{c}}^{updated}$ is met. Otherwise, keep $\theta_{k_{c}}$ as the current value.

**Algorithm 4** ISEM Algorithm1: Initialize the algorithm with a configuration $c^{\left(0\right)}=\left(c_{1}^{\left(0\right)},\ldots ,c_{n}^{\left(0\right)}\right)$ for allocation indicator variables.2: **procedure** For the *s*-th iteration of the algorithm, s=1,…,S, do the following.3:     Conditional on $c^{\left(s-1\right)}$, update the parameters of the clusters according to algorithm 3;4:     Obtain a new configuration $c^{\left(s\right)}$ for the allocation of indicator variables using algorithm 2.

## 4. Simulation Study

In this section, we describe the results from a simulation study carried out to verify the performance of the proposed algorithm. To generate the artificial datasets, we considered univariate normal mixture models. We set up the number of clusters and parameter values according to the specified values in Table 2. We also fixed the sample size equal to n=200.

The procedure for generating the datasets is given by the following steps:

(i) For i=1,…,n, generate $U_{i}∼U\left(0,1\right)$; if $\sum_{j^{\prime }=1}^{j-1}w_{j}<u_{i}\leq \sum_{j^{\prime }=1}^{j}w_{j}$, generate $Y_{i}∼N\left(\mu_{j},\sigma_{j}^{2}\right)$, with fixed parameter values according to Table 2, for $w_{0}=0$ and $j=1,\ldots ,k_{c}$.
(ii) In order to record from which component each observation is generated, we define $G=(G_{1},\ldots ,G_{n})$ such that $G_{i}=j$ if $Y_{i}∼N\left(\mu_{j},\sigma_{j}^{2}\right)$, for i=1,…,n and $j=1,\ldots ,k_{c}$.

Having generated the datasets, we need to define the the probability of creating a new cluster and the posterior distribution for $\theta_{j^{*}}=\left(\mu_{j^{*}},\sigma_{j^{*}}^{2}\right)$ given $y_{i}$, for i=1,…,n. For this, consider the following conjugated prior distributions for component parameters $\theta_{j}=\left(\mu_{j},\sigma_{j}^{2}\right)$:

$$\mu_{j}\left|\sigma_{j}^{2},\mu_{0},\lambda ∼N\left(\mu_{0},\frac{\sigma_{j}^{2}}{\lambda }\right)\;\;\;\;\;\mathrm{and}\;\;\;\;\;\sigma_{j}^{-2}\right|\alpha ,\beta ∼\Gamma \left(\alpha ,\beta \right),$$

where $\mu_{0}$, λ, α, and β are hyperparameters. The parametrization of the gamma distribution is such that the mean is α/β and the variance is $\alpha /\beta^{2}$.

Following [11,24], we consider the following procedure to define the values for the hyperparameters. Let *R* be the observed variation interval of the data and ε its midpoint. Then, we set up $\mu_{0}=\varepsilon$ and $E\left(\sigma_{j}^{-2}\right)=R^{-2}$. Thus, we obtain $\beta =\alpha R^{2}$, and we fix α=1. In addition, to obtain a prior distribution with a large variance, we fixed $\lambda =10^{-2}$, and for the hyperparameter γ, we consider the value 0.1, γ=0.1.

Thus, the probability of creating a new cluster is given by Equation (10), in which

(11) $$I\left(y_{i}\right)={\left[\frac{\lambda }{2\beta \pi (1+\lambda )}\right]}^{\frac{1}{2}}\frac{\Gamma (\alpha +1)}{\Gamma (\alpha )}{\left[1+\frac{y_{i}^{2}+\lambda \mu_{0}^{2}}{2\beta }-\frac{{\left(y_{i}+\lambda \mu_{0}\right)}^{2}}{2\beta (1+\lambda )}\right]}^{-(\alpha +\frac{1}{2})},$$

and $j^{*}=k_{c}+1$, for i=1,…,n.

When a new cluster is created, the new parameter values $\theta_{j^{*}}=\left(\mu_{j^{*}},\sigma_{j^{*}}^{2}\right)$ are generated from the following conditional posterior distributions,

(12) $$\begin{array}{c} \mu_{j^{*}}|\sigma_{j^{*}}^{2},y_{i},c,\mu_{0},\lambda ∼N\left(\frac{y_{i}+\lambda \mu_{0}}{1+\lambda },\frac{\sigma_{j}^{2}}{1+\lambda }\right) \end{array}$$

and

(13) $$\begin{array}{c} \sigma_{j^{*}}^{-2}|y_{i},c,\tau ,\beta ∼\Gamma \left(\alpha +1,\beta +\frac{y_{i}^{2}+\lambda \mu_{0}^{2}}{2}-\frac{{\left(y_{i}+\lambda \mu_{0}\right)}^{2}}{2(1+\lambda )}\right), \end{array}$$

for $j^{*}=k_{c_{-i}}+1$.

We run the ISEM algorithm for *S* = 55,000, *B* = 5000, and *h* = 10. From these values, we got a sub-sequence S(H) of size 5000 to make inferences. The algorithm was initialized with $k_{c}=1$ and parameter values $\mu_{1}=\bar{y}$ and $\sigma_{1}^{2}=s^{2}$, the sample mean and variance of the generated dataset, respectively.

We also apply to the generated datasets the SEM algorithm, as describe in Section 2, and the RJ algorithm as proposed by [8]. In order to choose the number of clusters using the SEM algorithm, we consider the AIC and BIC model selection criteria. In addition, the algorithm was initialized using a configuration $c^{\left(0\right)}$ obtained via the *k*-means algorithm [25]. As stop criterion, we set up the threshold ε=0.001. For the RJ algorithm, we consider the same number of iterations, burn-in, and thin value used in the ISEM algorithm.

In order to compare the three algorithms in terms of the estimation of the number of clusters, we consider M=500 simulated datasets. Table 3 shows the proportion of times that the ISEM and RJ algorithms put the highest estimated probability on the $k_{c}$ values presented. This table also show the proportion of times that the AIC and BIC indicated the $k_{c}$ value as the best among the tested values. The values highlighted in bold are the proportions on the $k_{c}$ true value. As one can note, the ISEM shows a better performance, i.e., higher proportion of the $k_{c}$ true value than the other two algorithms, especially in relation to the SEM algorithm with the selection of $k_{c}$ via the AIC and BIC. The results also show that the AIC and BIC model selection criteria have a low success ratio, with a proportion of the $k_{c}$ true value smaller than 0.50.

### 4.1. Results from a Single Simulated Data Set

We also analyze the results from a single dataset selected at random from the M=500 generated datasets in each situation $A_{1}$ to $A_{4}$. Then, we discuss the convergence of the ISEM and RJ algorithms based on the sample generated across iterations, using graphical tools. In general, the graphical tools show whether the simulated chain stabilizes in some sense and provide useful feedback about the convergence [26].

Table 4 shows the estimated probabilities of $k_{c}$ obtained with ISEM and RJ and the AIC and BIC values from the SEM algorithm for the selected dataset. In this table, the values highlighted in bold are the highest estimated probabilities and the smallest AIC and BIC values. As we can note, the ISEM algorithm set up a maximum probability for the $k_{c}$ true value for the four simulated cases.

The RJ algorithm puts a higher probability on the $k_{c}$ true value for datasets $A_{1}$ and $A_{2}$. However, the probability on the $k_{c}$ true value is smaller than that estimated by ISEM. This indicates a higher precision for the ISEM algorithm. For datasets $A_{3}$ and $A_{4}$, the RJ attributes maximum probability to the wrong values, $k_{c}=5$ and $k_{c}=6$, respectively. Moreover, the probabilities estimated by RJ do not evidence a single value for $k_{c}$ as being the best value since there are different values for $k_{c}$ with similar probabilities. For example, for dataset $A_{2}$, the maximum is at $k_{c}=3$ with $P(k_{c}=3|\cdot )=0.3836$, but one can argue that the estimated probabilities favor $k_{c}=3$ or $k_{c}=4$. For dataset $A_{3}$, there is similar support for $k_{c}$ between 4 and 7, and for $A_{4}$ between 5 and 7.

Analogously to ISEM and RJ, the AIC and BIC model selection criteria indicate the $k_{c}$ true value as the best value for datasets $A_{1}$ and $A_{2}$. For dataset $A_{3}$, similar to the RJ, the AIC indicates the wrong value $k_{c}=5$ as the best value, while the BIC indicates the $k_{c}$ true value as the best value. For dataset $A_{4}$, the AIC and BIC indicate the wrong value $k_{c}=6$ as the best model.

### 4.2. An Empirical Check of the Convergence

We now empirically check the convergence of the sequence of the probability for $k_{c}$ across iterations, the capacity to move for different values of $k_{c}$ in the course of the iterations, and the estimated autocorrelation function the (acf) for the ISEM and RJ algorithms.

**Figure 1:**
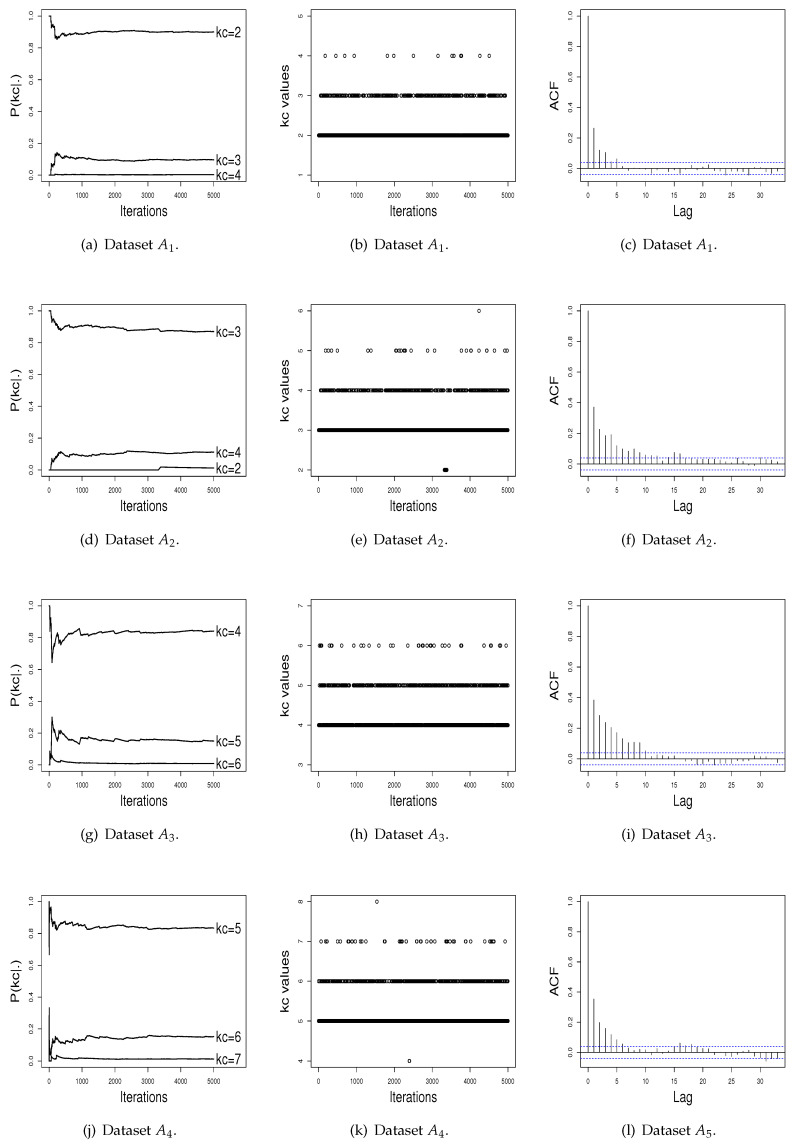
Performance of the ISEM algorithm across iterations.

Figure 1a,d,g,j presents the graphics of the probability for $k_{c}$ in the course of the iterations, for the four simulated datasets. To maintain a better visualization, we plot in these graphics only the three higher $P(k_{c}|\cdot )$ estimates. Observing at these figures, it can be seen that the L iterations and the burn-in value *B* used were adequate to achieve stability for $P(k_{c}|\cdot )$. In addition, Figure 1b,e,h,k shows that the ISEM algorithm mixes well over $k_{c}$, i.e., “visits” mixture models with different values of $k_{c}$ across iterations. As shown by Figure 1c,f,i,l, the sampled $k_{c}$ values also do not have significant autocorrelation function (ACF). Thus, based on these graphical tools, there is no evidence against the convergence of the generated values by the ISEM algorithm.

**Figure 2:**
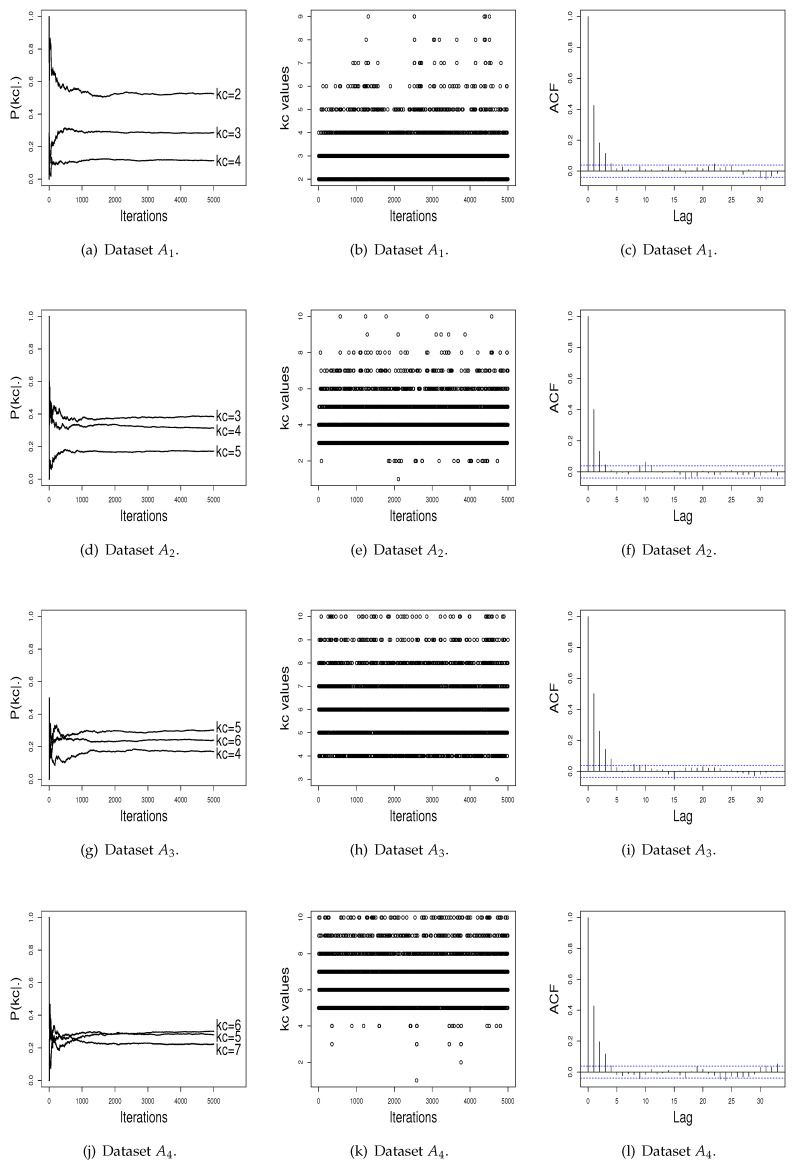
Performance of the RJ algorithm across iterations.

Figure 2 shows the performance of the RJ algorithm. The probabilities of $k_{c}$ present a satisfactory stability. The sampled $k_{c}$ values have a satisfactory mix, and the estimated autocorrelation is non-significant. In addition, as can be noted in Figure 2, probabilities for the number of clusters do not differentiate a value of $k_{c}$ in order to be chosen as the better value, as done by ISEM. This may happen due the fact that the performance of the RJ depends on the choice of the transition functions to do “good” jumping, meaning that a transition function that is adequate for one dataset may be not for another one. As the ISEM algorithm does not need the specification of transition functions to propose a change of the $k_{c}$ value, these results shows us that ISEM may be an effective alternative in relation to RJ and SEM algorithms for the joint estimation of $k_{c}$ and the cluster parameters of a mixture model.

Figure 1 in Appendix 2 of the SM shows the generated values for datasets $A_{1}$ to $A_{4}$. This Figure also shows the clusters identified by the ISEM algorithm. As can be seen, clusters are satisfactorily identified by the proposed algorithm.

We also compare ISEM and RJ algorithms in terms of CPU computation time. The simulations were realized on a MacBook Pro, 2.5 GHz Intel Core i5 dual core, 4 Gb MHz DDR3. Table 5 shows a summary of the times of iterations for the ISEM and RJ algorithms. The column denoted by s.d. presents the standard deviation values. For dataset $A_{1}$, the average time that RJ takes to run one iteration is 1.8491 times greater than the average time that ISEM takes to run an iteration. For datasets $A_{2}$, $A_{3}$, and $A_{4}$, the average time that RJ needs to run one iteration is 1.8175, 2.3239, and 1.8932 times greater than the average time that ISEM takes to run an iteration, respectively. These results show a better performance of the ISEM algorithm. The higher iteration times of the RJ algorithm are mainly due to the split–merge step used to increase the mixing of the Markov chain in relation to the number of clusters.

The results from these simulated datasets show that the ISEM algorithm may be an effective alternative to the RJ and SEM algorithms for data clustering in situations where the number of clusters is a unknown quantity.

## 5. Application

The three algorithms are now applied to two real datasets. The first real dataset refers to velocity in km/s of n=82 galaxies from 6 well-separated conic sections of an unfilled survey of the Corona Borealis region. This dataset is known in the literature as the Galaxy data and has already been analyzed by [8,13,22,27], among others. This dataset is available in the R software. The second real dataset refers to an acidity index measured in a sample of n=155 lakes in central-north Wisconsin. This dataset was downloaded from the website https://people.maths.bris.ac.uk/∼mapjg/mixdata.

For application of ISEM and RJ algorithms, we consider the same number *L* = 5500, *B* = 5000, and *h* = 10. Table 6 shows the estimated probabilities for $k_{c}$ obtained with ISEM and RJ and the AIC and BIC values from EM algorithm for each dataset. The maximum probability from ISEM and RJ and the minimum AIC and BIC values are highlighted in bold.

For the Galaxy dataset, the ISEM and RJ algorithms put highest probability on $k_{c}=3$ and $k_{c}=5$, respectively. However, analogously to the simulation study, the probabilities estimated by RJ do not evidence a single value for $k_{c}$ as being the best value. For this dataset, the estimated probabilities indicate a $k_{c}$ value between 3 and 7. The AIC and BIC also indicate $k_{c}=5$ as the best value. For the Acidity dataset, ISEM, AIC, and BIC indicate $k_{c}=2$ as the best value. The probabilities estimated by RJ attribute similar values for $k_{c}=3$ and $k_{c}=4$.

**Figure 3:**
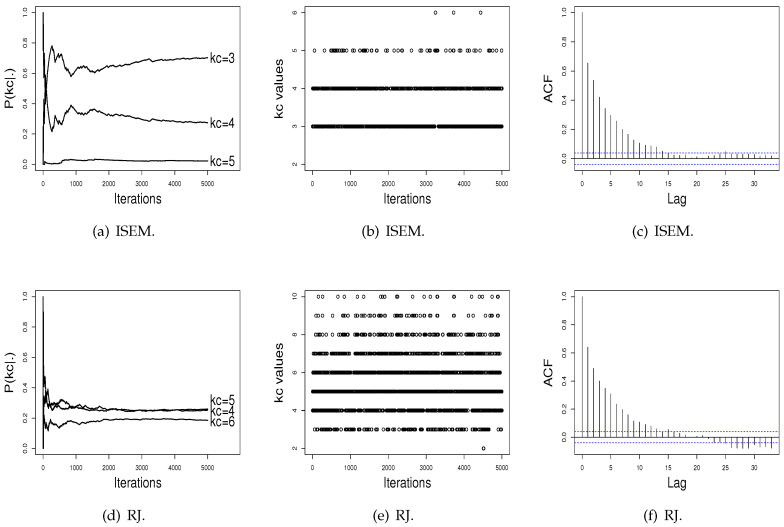
Performance of the ISEM and RJ algorithms for the Galaxy data.

**Figure 4:**
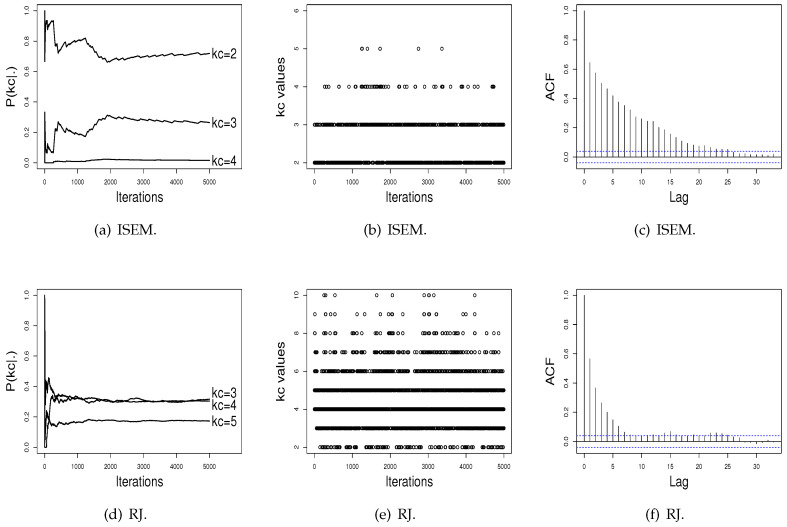
Performance of the RJ algorithm across iterations for the Acidity data.

Figure 3 and Figure 4 show the performance of the ISEM and RJ algorithms across iterations for the Galaxy and Acidity datasets. The values sampled by the ISEM algorithm present satisfactory stability for estimated probability across iterations, mix well among different $k_{c}$ values, and present no significant autocorrelation. That is, we do not have evidence against the convergence of the generated chain by the ISEM algorithm. In relation to the RJ, the sampled values mix well and do not present significant autocorrelation. However, although the values sampled by RJ present stability for $P(k_{c})$, the estimated probabilities do not differentiate a value of $k_{c}$ in order to be chosen as the better value, as done by ISEM. This result shows the need to run RJ for a greater number of iterations. With this, we have that for both real datasets, ISEM presents faster convergence than the RJ algorithm.

Table 7 shows a summary of the iteration times for the ISEM and RJ algorithms. For the Galaxy data, the average time that ISEM takes to run an iteration is 0.0053 s; while the average time for RJ is 0.0098 s. That is, the average time that RJ takes to run one iteration is 1.8491 times greater than the average time that ISEM takes to run an iteration. For the Acidity data, the average times that the ISEM and RJ algorithms take to run an iteration are 0.0085 and 0.0180 s, respectively. For this dataset, the average time that RJ needs to run an iteration is 2.2118 times greater than the average time that ISEM runs. Similarly to results from the simulation study, ISEM presents better results, i.e., a shorter time to run the iterations.

## 6. Final Remarks

This article presents a discussion of how to estimate the parameters of a mixture model in the context of data clustering. We propose an alternative algorithm to the EM algorithm called ISEM. This algorithm was developed through an integrated approach in order to allow $k_{c}$ to be estimated jointly with the other parameters of interest. In the ISEM algorithm, the allocation probabilities depend on the number of clusters $k_{c}$ and are independent of the number of components *k* of the mixture model.

In addition, there exists a positive probability of a new cluster being created by a single observation. This is an advantage of the algorithm because it creates a new cluster without the need to specify transition functions. In addition, the cluster parameters are updated according to the number of allocated observations. For the clusters with at least two of these observations, the values of the parameters are taken by the maximum likelihood estimates. For a cluster with just one observation, the parameter values are generated from the posterior distribution.

In order to illustrate the performance of the ISEM algorithm, we developed a simulation study. In this simulation study, we considered four scenarios with artificial data generated from Gaussian mixture models. In addition, each one of the four scenarios was replicated M=500 times, and the proportion of times that ISEM put a higher probability on the $k_{c}$ true value was recorded. We applied this same procedure to the EM algorithm, choosing the number of clusters $k_{c}$ via the AIC and BIC, and to the RJ algorithm. Then, the three algorithms were compared in terms of proportion of times that the $k_{c}$ true value was selected as the best value. The results obtained show that the ISEM algorithm outperforms the RJ and SEM algorithms. Moreover, the results also show that the AIC and BIC model selection criteria should not be used to determine the number of clusters in a mixture model due to a low success rate.

We also compared the performance of ISEM and RJ in terms of empirical convergence of the sequence of values generated using graphical tools. For this, we selected at random an artificial dataset from each scenery, and then we plotted the probability estimates for $k_{c}$ across iterations, the generated $k_{c}$ values, and the estimated autocorrelation of the sampled values (see Figure 1 and Figure 2). Again, the results show a better performance for the ISEM algorithm. While ISEM presents satisfactory stability for the probability of $k_{c}$ and differentiates the true $k_{c}$ as the best value, the probabilities estimated by RJ do not differentiate a value of $k_{c}$ in order to be chosen as the better value.

In order to illustrate the practical use of the proposed algorithm and compare its performance with the SEM and RJ algorithms, we applied the three algorithms to two real datasets: the Galaxy and Acidity datasets. For the Galaxy dataset, ISEM indicates $k_{c}=3$ with probability $P(k_{c}=3|\cdot )=0.7024$, while the RJ algorithm, the AIC, and the BIC indicate $k_{c}=5$. However, as shown in Figure 3d, the RJ algorithm again does not differentiate a value of $k_{c}$, while ISEM differentiates the $k_{c}=3$ value, and the generated values across iterations present satisfactory stability. For the Acidity dataset, the ISEM, AIC, and BIC indicate $k_{c}=2$ as the best value, while RJ attributes similar probabilities for $k_{c}=3$ and $k_{c}=4$.

As mentioned in the Introduction, the generalization of the proposed algorithm for the multivariate case is the next step of our research. The simulation study and the application were done in R software, and the computational codes can be obtained by emailing the authors.

## Figures and Tables

**Table 1:** Main mathematical notation used throughout the paper.

Notation	Description
*k*	Number of components
$k_{c}$	Number of clusters
$\theta_{j}$	Parameter of the *j*-th component, for j=1,…,k
$\theta_{k}=\left(\theta_{1},\ldots ,\theta_{k}\right)$	The whole vector of parameters
$w_{j}$	Weight of the *j*-th component, for j=1,…,k
$Y_{i}$	The *i*-th sampled value, for i=1,…,n
$c_{i}$	The *i*-th indicator variable, for i=1,…,n
$y=(y_{1},\ldots ,y_{n})$	The vector of independent observations
$c=(c_{1},\ldots ,c_{n})$	The vector of latent indicator variables
$k_{c_{-i}}$	Number of clusters excluding the *i*-th observation
$n_{j,-i}$	Number of observations assigned to the *j*-th component, excluding the *i*-th observation

**Table 2:** Number of clusters and parameter values used for simulating the datasets.

Artificial Dataset	Number of Clusters	Parameter Values				
$A_{1}$	$k_{c}=2$	$\mu_{1}=0$,	$\mu_{2}=3$,			
		$\sigma_{1}^{2}=1$,	$\sigma_{2}^{2}=1$,			
		$w_{1}=0.80$,	$w_{2}=0.20$,			
$A_{2}$	$k_{c}=3$	$\mu_{1}=-6$,	$\mu_{2}=0$,	$\mu_{3}=4$		
		$\sigma_{1}^{2}=3$,	$\sigma_{2}^{2}=2$,	$\sigma_{3}^{2}=1$		
		$w_{1}=0.50$,	$w_{2}=0.30$,	$w_{3}=0.20$		
$A_{3}$	$k_{c}=4$	$\mu_{1}=-6$,	$\mu_{2}=0$,	$\mu_{3}=7$,	$\mu_{4}=14$	
		$\sigma_{1}^{2}=1$,	$\sigma_{2}^{2}=2$,	$\sigma_{3}^{2}=2$,	$\sigma_{4}^{2}=1$	
		$w_{1}=0.10$,	$w_{2}=0.40$,	$w_{3}=0.40$,	$w_{4}=0.10$	
$A_{4}$	$k_{c}=5$	$\mu_{1}=-13$,	$\mu_{2}=7$,	$\mu_{3}=0$,	$\mu_{4}=6$,	$\mu_{5}=11$
		$\sigma_{1}=1$,	$\sigma_{2}=2$,	$\sigma_{3}=3$,	$\sigma_{4}=2$,	$\sigma_{5}=1$
		$w_{1}=0.15$,	$w_{2}=0.20$,	$w_{3}=0.30$,	$w_{4}=0.20$,	$w_{5}=0.15$,

**Table 3:** Proportion of times the algorithms chose the $k_{c}$ values as the number of clusters.

Data Set	$k_{c}^{\mathrm{true}}$	$k_{c}$	$\tilde{P}\left(k_{c}=j\cdot \right)$		AIC	BIC	Data Set	$k_{c}^{\mathrm{true}}$	$k_{c}$	$\tilde{P}\left(k_{c}=j\cdot \right)$		AIC	BIC
			ISEM	RJ						ISEM	RJ		
$A_{1}$	2	1	0.014	0.002	0.050	0.210	$A_{2}$	3	1	0.000	0.000	0.000	0.004
		2	**0.976**	**0.972**	**0.294**	**0.448**			2	0.276	0.094	0.104	0.438
		3	0.010	0.026	0.238	0.224			3	**0.720**	**0.672**	**0.304**	**0.384**
		4	0.000	0.000	0.152	0.082			4	0.004	0.232	0.262	0.138
		5	0.000	0.000	0.148	0.028			5	0.000	0.002	0.184	0.028
		6	0.000	0.000	0.118	0.008			6	0.000	0.000	0.146	0.008
$A_{3}$	4	1	0.000	0.000	0.000	0.000	$A_{4}$	5	1	0.000	0.000	0.000	0.000
		2	0.000	0.004	0.000	0.000			2	0.006	0.000	0.000	0.006
		3	0.000	0.000	0.010	0.066			3	0.006	0.000	0.000	0.018
		4	**0.956**	**0.476**	**0.226**	**0.450**			4	0.218	0.010	0.038	0.210
		5	0.044	0.474	0.252	0.296			5	**0.682**	**0.509**	**0.322**	**0.446**
		6	0.000	0.044	0.214	0.122			6	0.028	0.442	0.246	0.222
		7	0.000	0.000	0.184	0.056			7	0.000	0.039	0.210	0.072
		8	0.000	0.002	0.114	0.010			8	0.000	0.000	0.184	0.026

**Table 4:** Estimated probability for $k_{c}$.

Data Set	$k_{c}^{\mathrm{true}}$	$k_{c}$	$\tilde{P}\left(k_{c}=j\cdot \right)$		AIC	BIC	Data Set	$k_{c}^{\mathrm{true}}$	$k_{c}$	$\tilde{P}\left(k_{c}=j\cdot \right)$		AIC	BIC
			ISEM	RJ						ISEM	RJ		
$A_{1}$	2	1	0.0000	0.0000	786.7166	793.3133	$A_{2}$	3	1	0.0000	0.0004	1160.758	1167.355
		2	**0.9006**	**0.5252**	**762.5204**	**779.0120**			2	0.0122	0.0136	1129.981	1146.472
		3	0.0962	0.2862	764.1440	790.5305			3	**0.8694**	**0.3836**	**1114.024**	**1140.411**
		4	0.0032	0.1138	769.2648	805.5463			4	0.1124	0.3140	1118.789	1155.070
		5	0.0000	0.0466	768.0492	814.2256			5	0.0058	0.1716	1120.108	1166.284
		6	0.0000	0.0160	775.1082	831.1796			6	0.0002	0.0744	1130.558	1186.630
		≥7	0.0000	0.0122	-	-			≥7	0.0000	0.0424	-	-
$A_{3}$	4	1	0.0000	0.0000	1273.886	1280.482	$A_{4}$	5	1	0.0000	0.0002	1416.124	1422.721
		2	0.0000	0.0000	1276.281	1292.773			2	0.0000	0.0004	1388.738	1405.230
		3	0.0000	0.0002	1251.357	1277.743			3	0.0000	0.0028	1358.474	1384.861
		4	**0.8412**	0.1696	1188.470	**1224.751**			4	0.0014	0.0114	1357.037	1393.318
		5	0.1500	**0.3014**	**1186.075**	1232.252			5	**0.8340**	0.2788	1355.922	1402.098
		6	0.0088	0.2400	1191.747	1247.818			6	0.1520	**0.3004**	**1325.927**	**1381.998**
		7	0.0000	0.1632	1197.028	1262.995			7	0.0124	0.2224	1331.940	1397.907
		8	0.0000	0.0816	1200.337	1276.199			8	0.0002	0.0186	1331.352	1407.213
		≥9	0.0000	0.0440	-	-			≥9	0.0000	0.0750	-	-

**Table 5:** Times of the iterations, in seconds.

Artificial Dataset	Algorithm	Summary						
		Min	1^o^ Q.	Med.	Mean	3^o^ Q.	Max.	s.d.
$A_{1}$	ISEM	0.0064	0.0082	0.0091	0.0109	0.0105	0.4987	0.0107
	RJ	0.0032	0.0137	0.0158	0.0208	0.0202	0.3855	0.0174
$A_{2}$	ISEM	0.0055	0.0100	0.0114	0.0137	0.0146	0.3806	0.0108
	RJ	0.0032	0.0169	0.0196	0.0249	0.0243	0.7709	0.0181
$A_{3}$	ISEM	0.0059	0.0112	0.0123	0.0142	0.0139	0.4951	0.0100
	RJ	0.0020	0.0218	0.0255	0.0330	0.0320	0.4785	0.0239
$A_{4}$	ISEM	0.0059	0.0130	0.0146	0.0179	0.0187	0.5149	0.0108
	RJ	0.0026	0.0232	0.0266	0.0339	0.0323	0.5490	0.0231

**Table 6:** Estimated probabilities for $k_{c}$, real datasets.

Data Set	$k_{c}$	$\tilde{P}\left(k_{c}=j\cdot \right)$		AIC	BIC	Data Set	$k_{c}$	$\tilde{P}\left(k_{c}=j\cdot \right)$		AIC	BIC
		ISEM	RJ					ISEM	RJ		
Galaxy	1	0.0000	0.0000	484.6819	489.4954	Acidity	1	0.0000	0.0000	455.5740	461.6608
	2	0.0000	0.0008	451.0018	463.0354		2	**0.7194**	0.0502	**380.3449**	**395.5620**
	3	**0.7024**	0.1200	426.7421	445.09959		3	0.2638	**0.3164**	382.7395	407.0869
	4	0.2748	0.2530	427.4915	453.9654		4	0.0152	0.3040	382.3660	415.8437
	5	0.0222	**0.2592**	**410.3666**	**444.0607**		5	0.0016	0.1724	391.7630	434.3709
	6	0.0006	0.1848	413.7755	454.6897		6	0.0000	0.0832	386.1420	437.8802
	7	0.0000	0.1084	422.1793	470.3137		7	0.0000	0.0452	388.1296	448.9981
	8	0.0000	0.0472	423.5542	478.9088		8	0.0000	0.0186	395.3957	465.3945
	≥9	0.0000	00226	-	-		≥9	0.0000	0.0010	-	-

**Table 7:** Iteration times in seconds.

Artificial Dataset	Algorithm	Summary						
		Min	1^o^ Q.	Med.	Mean	3^o^ Q.	Max.	s.d.
Galaxy	ISEM	0.0023	0.0038	0.0045	0.0053	0.0054	0.2468	0.0062
	RJ	0.0000	0.0000	0.0000	0.0000	0.0000	0.0000	0.0000
Acidity	ISEM	0.0055	0.0100	0.0114	0.0137	0.0146	0.3806	0.0108
	RJ	0.0046	0.0128	0.0149	0.0188	0.0180	0.4588	0.0160

## Supplementary Materials

The following are available online at https://www.mdpi.com/1099-4300/21/11/1063/s1.
