# Supplementary material for: An Integrated Approach for Making Inference on the Number of Clusters in a Mixture Model
Source: Entropy (Basel). 2019 Oct 30;21(11):1063. doi: 10.3390/e21111063 (PMC7514367; doi:10.3390/e21111063)
Supplement: Supplementary file 1 [file entropy-21-01063-s001.zip › SM_Entropy-611769/SM_ISEM.pdf]

Supplementary Material of the paper: An Integrated Approach for Making Inference on the Number of Clusters in a Mixture Model

Erlandson F. Saraiva, Adriano K. Suzuki, Luís A. Milan and Carlos A. B. Pereira

This supplementary material (SM) presents the criterion used to define a configuration for the latent allocation variables **c** and how we obtain the estimates for parameters of the clusters. Besides, we also show the graphics of the generated and identified clusters by proposed ISEM algorithm and estimates for parameters.

Appendix 1: Estimation

As decribed in page 7 of the paper, in order to estimate the number of clusters $k_{\mathbf{c}}$, we consider $\mathbb{N}_{k_{\mathbf{c}}}(j)$ be the number of times that $k_{\mathbf{c}} = j$ in the generated sequence $\mathbb{S}(H)$, for $j \in \{1, \ldots, K_{max}\}$, and calculate $N_{k=j}$, which denotes the number of times that $k_{\mathbf{c}} = j$ in $\mathbb{S}(H)$. Letting $\tilde{P}(k_{\mathbf{c}} = j) = \frac{\mathbb{N}_{k_{\mathbf{c}}}(j)}{H}$ be the posterior probability for $k_{\mathbf{c}} = j$, then $\widetilde{k}_{\mathbf{c}} = \arg\max\limits_{1 \leq j \leq k_m}(P(k = j|\cdot))$ is the estimate for the number of components.

Conditional on estimate $\widetilde{k}_{\mathbf{c}}$, consider

(i) $L_{\widetilde{k}_{\mathbf{c}}} = \sum\limits_{\mathbb{S}(H)} \mathcal{I}_{k_{\mathbf{c}}^{(s)}}\left(\widetilde{k}_{\mathbf{c}}\right)$, where $\mathcal{I}_{k_{\mathbf{c}}^{(s)}}(\widetilde{k}_{\mathbf{c}}) = 1$ if the s-th value of $\mathbb{S}(H)$ is $k_{\mathbf{c}} = \widetilde{k}_{\mathbf{c}}$ and $\mathcal{I}_{k_{\mathbf{c}}^{(s)}}(\widetilde{k}_{\mathbf{c}}) = 0$ otherwise, the number of times in which $k_{\mathbf{c}} = \widetilde{k}_{\mathbf{c}}$ in the sequence $\mathbb{S}(H)$;

(ii) $N_{ij} = \sum\limits_{\mathbb{S}(H)} \mathcal{I}_{c_i^{(s)}}(j) \mathcal{I}_{k_{\mathbf{c}}^{(s)}}\left(\widetilde{k}_{\mathbf{c}}\right)$, where $\mathcal{I}_{c_i^{(s)}}(j) = 1$ if in s-th iteration $c_i = j$ and $\mathcal{I}_{c_i^{(s)}}(j) = 0$ otherwise, the number of times that y_i is associated to component j in $L_{\widetilde{k}_{\mathbf{c}}}$ iterations, $i = 1, \ldots, n$ and $j = 1, \ldots, \widetilde{k}_{\mathbf{c}}$.

Then, we define the posterior probability of the observation y_i to be from component j as $P_{ij} = N_{ij}/L_{\widetilde{k}_{\mathbf{c}}}$. If $P_{ij} = \underset{1 \leq j \leq \widetilde{k}_{\mathbf{c}}}{max}(P_{ij})$, we consider that y_i is from component j, for $i = 1, \ldots, n$ and $j = 1, \ldots, \widetilde{k}_{\mathbf{c}}$.

We estimate the component parameter θ_j of the j-th cluster, for $j = 1, \ldots, \widetilde{k}_{\mathbf{c}}$, considering the average of the generated values, *i.e.*,

$$\widetilde{\theta}_j | \widetilde{k}_{\mathbf{c}} = \frac{1}{L_{\widetilde{k}_{\mathbf{c}}}} \sum_{s=B+1}^{S} \phi_j^{(s)} \mathcal{I}_{k_{\mathbf{c}}^{(s)}}\left(\widetilde{k}_{\mathbf{c}}\right).$$

Appendix 2: Generated and identified clusters

In this section, we present some additional results from simulation study. Figure 1 shows the generated values and the identified clusters by the ISEM algorithm for datasets A_1 and A_2. Figure 2 shows the generated values and the identified clusters by the ISEM algorithm for datasets A_3 and A_4.

The clusters were defined acording to the procedure describe in Apendix 1. As one can note, clusters are satisfactorily identified by the proposed algorithm.

Table 1 shows the estimates for component parameters of the identified clusters and the empirical credibilities intervals (95%) for parameters of datasets A_1 and A_2. Figure 3 shows the histogram of the observed data and the estimated density function.

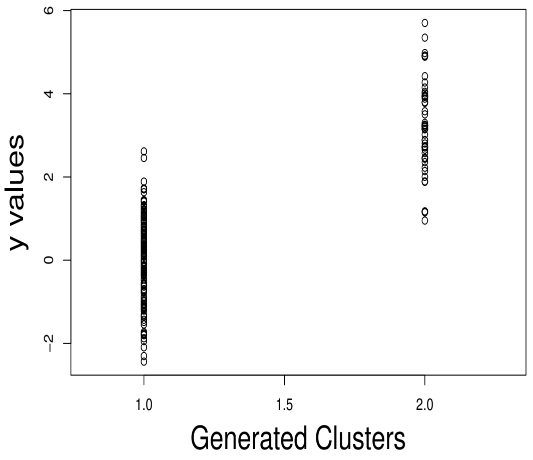

(a) Dataset A_1, Generated.

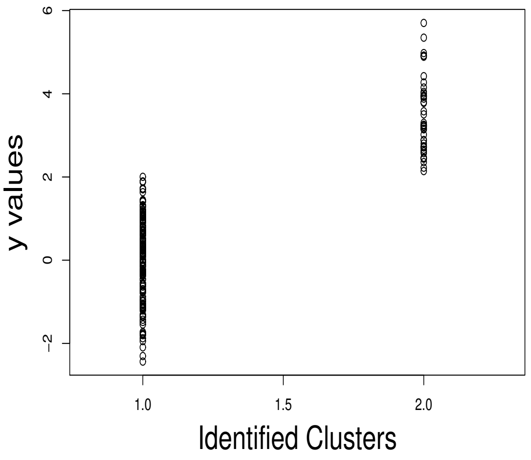

(b) Dataset A_1, Identified.

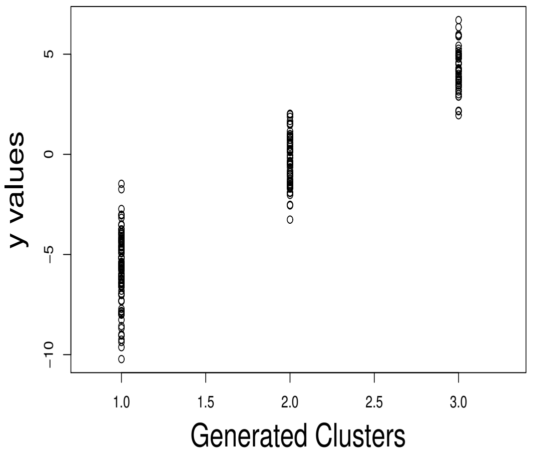

(c) Dataset A_2, Generated.

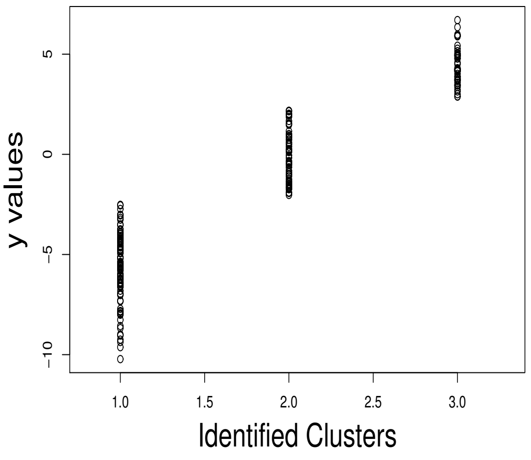

(d) Dataset A_2, Identified.

Figure 1: Generated values and the identified clusters by the ISEM algorithm.

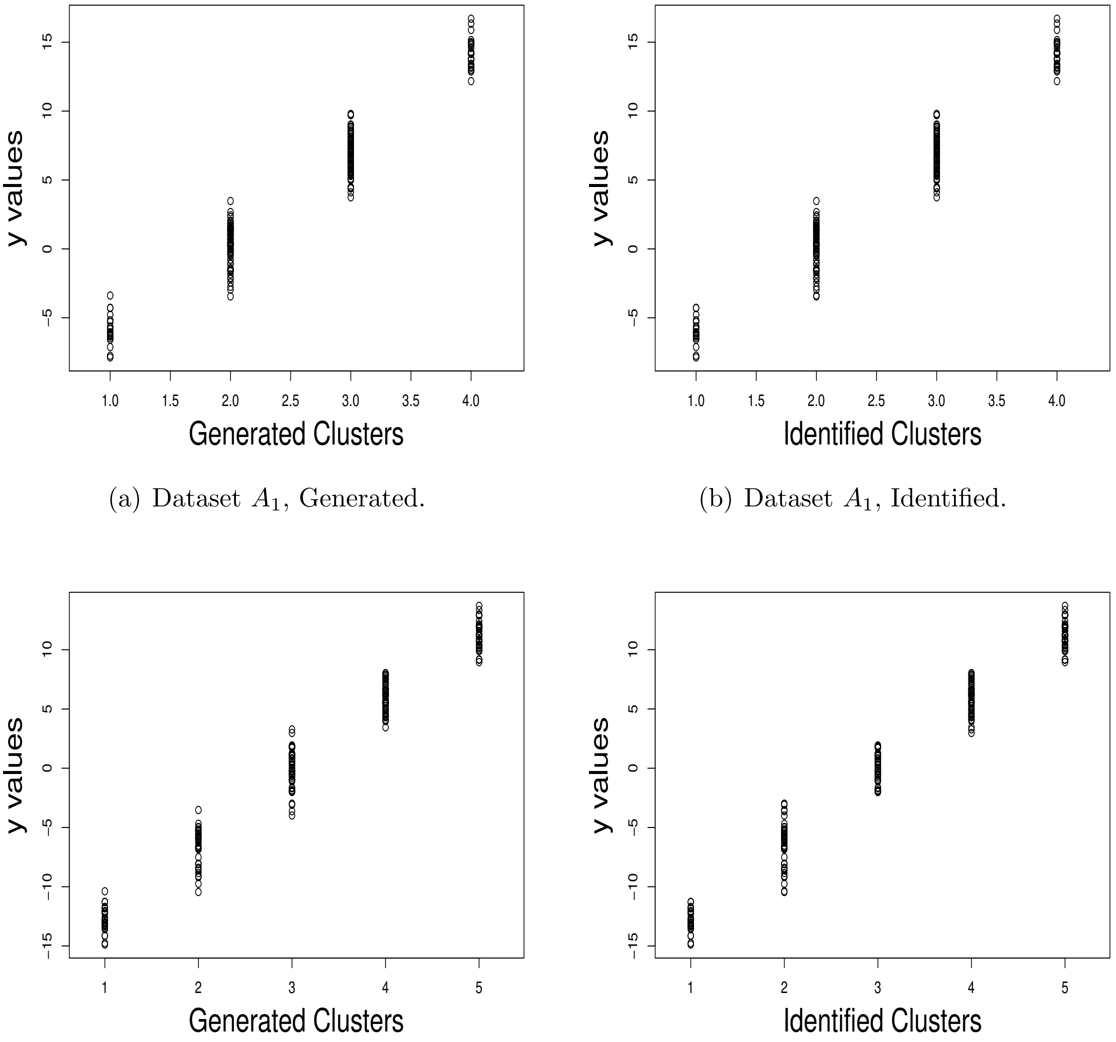

(a) Dataset A_1, Generated.

(b) Dataset A_1, Identified.

(c) Dataset A_2, Generated.

(d) Dataset A_2, Identified.

Figure 2: Generated values and the identified clusters by the ISEM algorithm.

Table 1: Estimates for component parameters of the $\tilde{k}_{\mathbf{c}}$ clusters.

Parameter	Data set		Parameter	Data set	
	A_1	A_2		A_1	A_2
μ_1	0.0892 $(-0.0810, 0.2360)$	-5.5705 $(-5.9938, -5.0868)$	σ_1^2	1.1111 $(0.9074, 1.3809)$	3.7208 $(2.7779, 5.1638)$
μ_2	3.2937 $(2.5563, 3.6751)$	-0.0560 $(-0.5754, 0.4957)$	σ_2^2	1.1876 $(0.7426, 2.2491)$	2.5742 $(1.1976, 6.70054)$
μ_3	$-$	4.3179 $(3.9722, 4.5729)$	σ_3^2	$-$	1.1118 $(0.7565, 1.7803)$

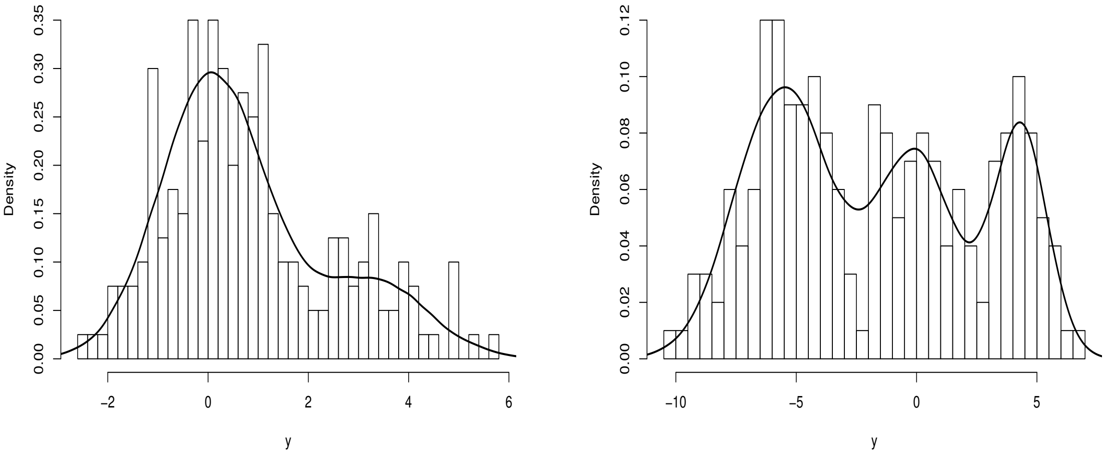

(a) Dataset A_1, Generated.

(b) Dataset A_1, Identified.

Figure 3: Histogram of generated dataset and estimated density for datasets A_1 and A_2.

Table 2 shows the estimates for component parameters of the identified clusters and the empirical credibilities intervals (95%) for parameters of datasets A_3 and A_4. Figure 4 shows the histogram of the observed data and the estimated density function.

Table 2: Estimates for component parameters of the $\tilde{k}_{\mathbf{c}}$ clusters.

Parameter	Data set		Parameter	Data set	
	A_3	A_4		A_3	A_4
μ_1	-5.8787 $(-6.1965, -5.2799)$	-12.8645 $(-13.0360, -12.7018)$	σ_1^2	1.2591 $(0.6456, 2.7882$	0.8654 $(0.5213, 1.2372$
μ_2	0.2999 $(0.1314, 0.5051$	-6.3791 $(-6.9645, -5.4280$	σ_2^2	2.3361 $(1.5482, 3.2709$	2.4554 $(1.6450, 4.1544$
μ_3	6.1970 $(6.8023, 7.0266)$	0.0975 $(-0.2085, 0.4903)$	σ_3^2	1.9360 $(1.7023, 2.2830)$	1.8476 $(1.0119, 3.0996$
μ_4	14.1870 $(14.1863, 14.1863)$	5.9537 $(5.7438, 6.1972)$	σ_4^2	1.2389 $(0.7386, 2.2870)$	1.9687 $(1.3533, 3.0779$
μ_5	$-$	11.2635 $(11.0470, 11.4631)$	σ_5^2	$-$	1.4249 $(1.0509, 1.9775$

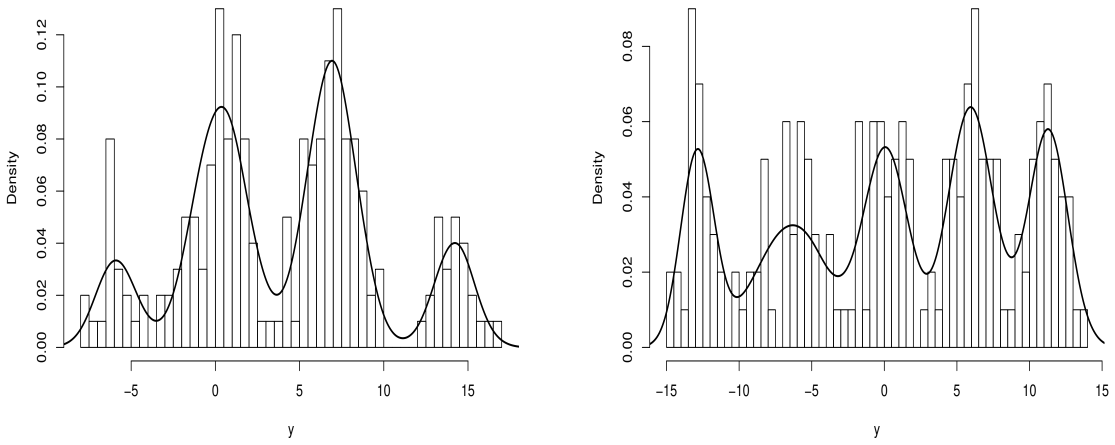

(a) Dataset A_1, Generated.

(b) Dataset A_1, Identified.

Figure 4: Histogram of generated dataset and estimated density for datasets A_1 and A_2.

Appendix 3: Some details on Equation 5

From Equation (4) of the manuscript, we have that

$$\pi(\mathbf{c}|\mathbf{w}, k) = \prod_{j=1}^{k} w_j^{n_j}, \tag{1}$$

where n_j is the number of observations allocated to j-th component.

As we assume that $\mathbf{w} = (w_1, \ldots, w_k)|\gamma, k \sim \textit{Dirichlet}\left(\frac{\gamma}{k}, \ldots, \frac{\gamma}{k}\right)$, then

$$\pi(\mathbf{w}|k, \gamma) = \frac{\Gamma(\gamma)}{\left[\Gamma\left(\frac{\gamma}{k}\right)\right]^k} \prod_{j=1}^{k} w_j^{\frac{\gamma}{k}-1}. \tag{2}$$

Thus,

$$\begin{aligned}
\pi(\mathbf{c}|k, \gamma) &= \int \ldots \int \pi(c_1, \ldots, c_k|\mathbf{w}, k)\pi(w_1, \ldots, w_k|\gamma, k) dw_1 \ldots dw_k \\
&= \frac{\Gamma(\gamma)}{\left[\Gamma\left(\frac{\gamma}{k}\right)\right]^k} \int \prod_{j=1}^{k} w_j^{n_j+\frac{\gamma}{k}-1} dw_j \\
&= \frac{\Gamma(\gamma)}{\left[\Gamma\left(\frac{\gamma}{k}\right)\right]^k} \frac{1}{n+\gamma} \prod_{j=1}^{k} \Gamma\left(n_j + \frac{\gamma}{k}\right). \\
&= \frac{\Gamma(\gamma)}{\Gamma\left(n+\gamma\right)} \prod_{j=1}^{k} \frac{\Gamma\left(n_j + \frac{\gamma}{k}\right)}{\Gamma\left(\frac{\gamma}{k}\right)}.
\end{aligned}$$
